# Supplementary material for: Maternal aggression driven by the transient mobilisation of a dormant hormone-sensitive circuit
Source: Nat Commun. 2025 Sep 29;16:8553. doi: 10.1038/s41467-025-64043-4 (PMC12480473; doi:10.1038/s41467-025-64043-4)
Supplement: Supplementary file 1 — Supplementary Information [file 41467_2025_64043_MOESM1_ESM.pdf]

Supplementary Information

Maternal Aggression Driven by the Transient Mobilisation of a Dormant Hormone-Sensitive Circuit

Stefanos Stagkourakis[1,], Paul Williams[2], Giada Spigolon[1], Shreya Khanal[2], Katharina Ziegler[2], Laura Heikkinen[2], Gilberto Fisone[1] and Christian Broberger[1,2,*]*

[1] SciLifeLab, Department of Neuroscience, Karolinska Institutet, Box 1031, SE-17121 Solna, Sweden

[2] Department of Biochemistry and Biophysics, Stockholm University, Svante Arrhenius väg 16C, 104 05 Stockholm, Sweden

*Correspondence: stefanos.stagkourakis@scilifelab.se, christian.broberger@dbb.su.se

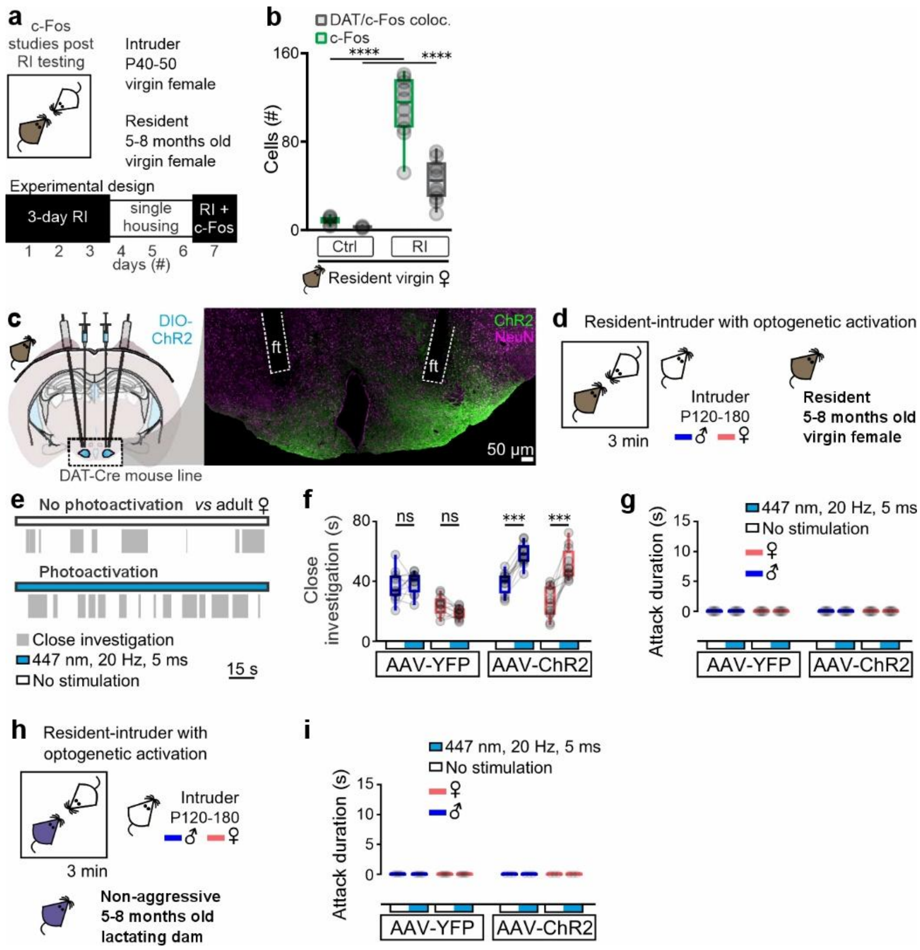

Supplemental Figure 1 | PMv^{DAT} neurons are activated following an encounter with a conspecific, and their photostimulation leads to increased social investigation but not aggression, in virgin female mice.

a Schematic representation of the experimental design used to perform c-Fos studies following RI testing.

b Quantification of c-Fos and DAT-tdTomato positive cells in control and social conditions, in virgin female mice in the estrus phase of the estrous cycle (n=8 sections from 4 mice without intruder exposure, and n = 8 sections from 4 mice with intruder exposure; comparisons performed using ordinary one-way ANOVA with Tukey's test for correcting for multiple comparisons, ANOVA P value<0.0001).

c Bilateral Cre-dependent ChR2 transduction and fiber implants in the PMv of virgin female DAT-Cre mice (left) and confocal image of a sample section stained with the pan-neuronal marker NeuN, used to validate ChR2 expression and optic fiber (ft) implantation coordinates (right).

d Schematic of the experimental design used to perform ChR2 PMv^{DAT} neuron photoactivation in the resident virgin female mouse during resident-intruder (RI) testing. Adult male and female mice were used as intruders.

e Sample behaviour raster plots at baseline (top) and during ChR2 stimulation (bottom) in a virgin female mouse during an RI test.

f Close investigation duration with and without photostimulation in virgin female mice injected with eYFP or ChR2, during the RI test against adult male or adult female intruders (n=12 mice per group, RM one-way ANOVA with Tukey's test for correcting for multiple comparisons, ANOVA P value<0.0001).

g Attack duration with and without photostimulation in virgin female mice injected with eYFP or ChR2, during the RI test against adult male or adult female intruders (n=12 mice per group, all values are zero).

h Schematic of the experimental design used to perform ChR2 PMv^{DAT} neuron photoactivation in the resident non-aggressive lactating dam during resident-intruder (RI) testing. Adult male and female mice were used as intruders.

i Attack duration with and without photostimulation in non-aggressive lactating dams injected with eYFP or ChR2, during the RI test against adult male or adult female intruders (n=5 mice in the eYFP and n=3 mice in the ChR2 group, all values are zero).

All box plots show the median (center line), 25th and 75th percentiles (box bounds), and minima/maxima (whiskers). Source data are provided as Source Data file.

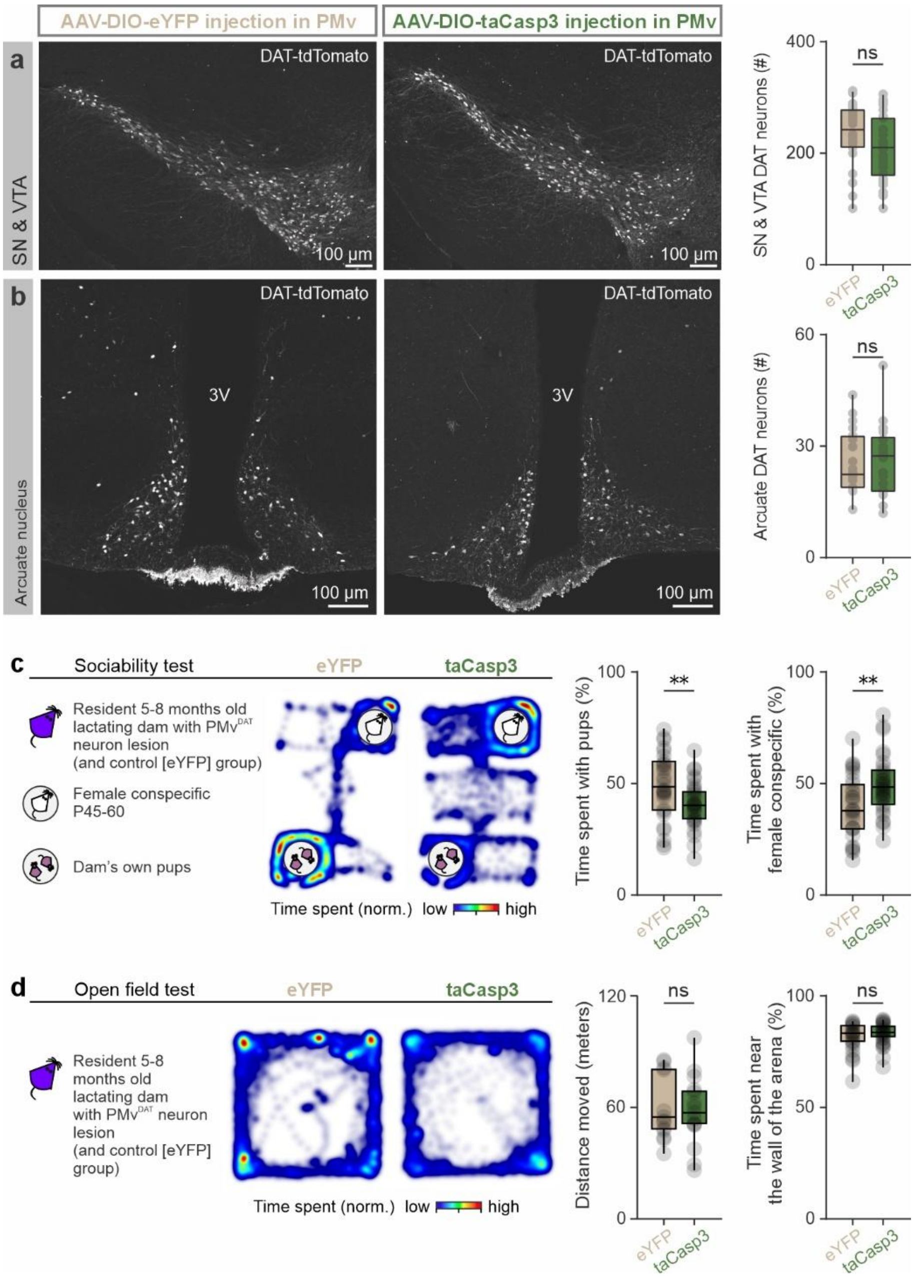

Supplemental Figure 2 | Injection of AAV-flex-taCasp3 in the PMv of DAT-Cre animals does not extend along the rostrocaudal axis, leaving the midbrain and arcuate nucleus dopamine neurons intact.

a Confocal image of an eYFP- (left) *vs.* taCasp3- (middle) PMv injected sample sections of midbrain (substantia nigra, SN, and ventral tegmental area, VTA) DAT-tdTomato neurons, and quantification of cell number per section (n=24-26 sections per group, duplicates from 12-13 mice per group, two-tailed unpaired t-test, P=0.1141).

b Confocal image of an eYFP- (left) *vs.* taCasp3- (middle) PMv injected sample sections of arcuate DAT-tdTomato neurons, and quantification of cell number per section (n=24-26 sections per group, duplicates from 12-13 mice per group, two-tailed unpaired t-test, P=0.5650). 3V, third ventricle.

c Design and results from the sociability test of lactating dams with complete or partial lesion of the PMv^{DAT} cells. Dams were given two options, one chamber containing a juvenile virgin female conspecific, and another chamber with three of the dam’s own pups (n=30-33 trials, duplicates or triplicates from 12 mice per group, two-tailed unpaired t-test, P=0.0068, and P=0.0032 respectively).

d Results from the open field test of lactating dams with complete or partial lesion of the PMv^{DAT} cells. None of the variables quantified were different among the two group (n=12 mice per group, two-tailed unpaired t-test, P=0.7422, and P=0.2346 respectively).

All box plots show the median (center line), 25th and 75th percentiles (box bounds), and minima/maxima (whiskers). Source data are provided as Source Data file.

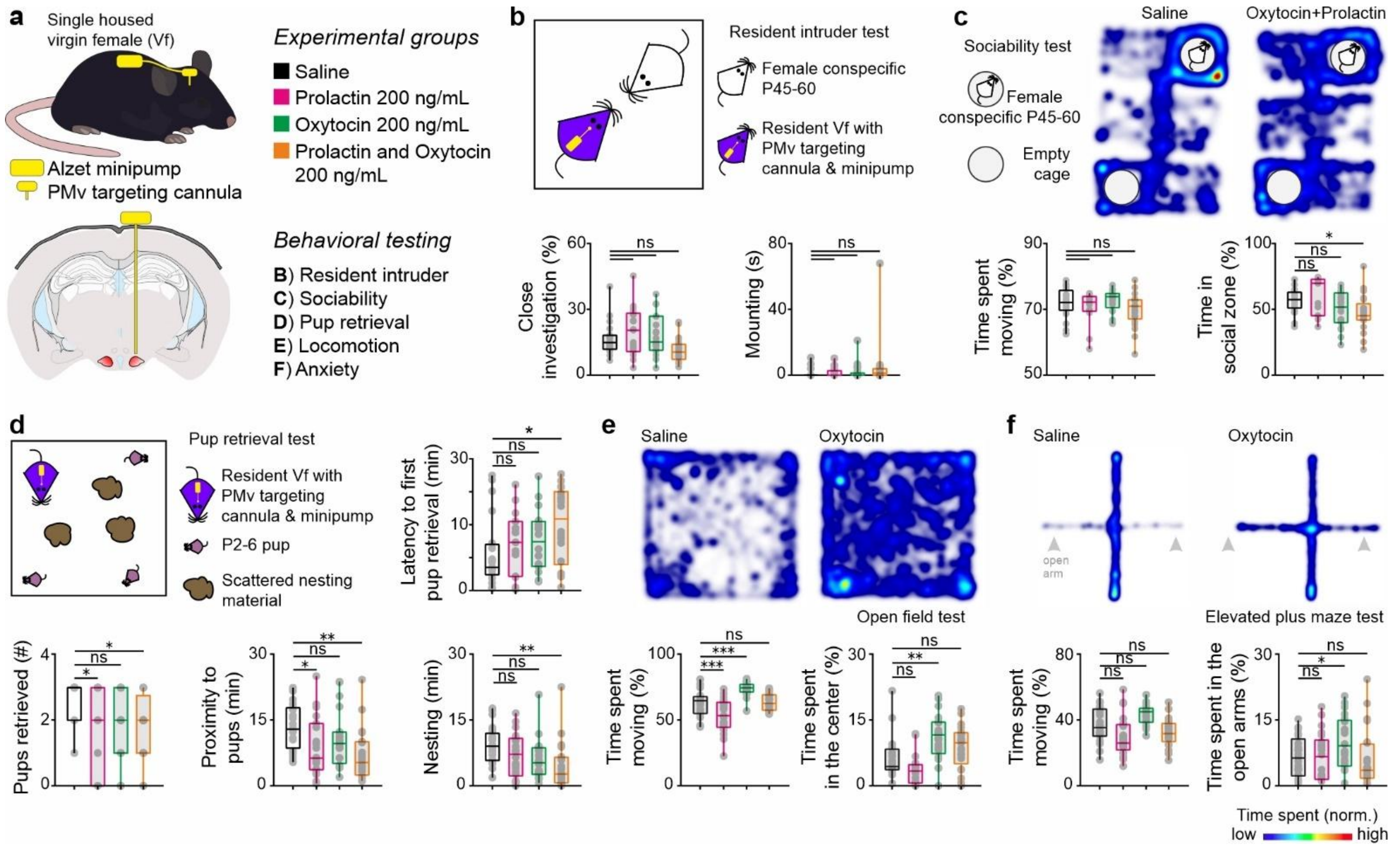

Supplemental Figure 3 | Chronic administration of maternal hormones in the PMv is not sufficient to evoke aggression in virgin females.

a Schematic of the experimental design followed to infuse maternal hormones into the PMv parenchyma via cannulas for a period of 28 days, and perform a panel of behavioural tests.

b Design and quantification of social behaviours in the RI test, with implanted adult virgin female residents *vs.* juvenile virgin female intruders. No changes in close investigation or mounting duration were recorded (n=21-24 trials per group, triplicates from 7-8 mice per group, RM one-way ANOVA with Tukey's test for correcting for multiple comparisons, ANOVA P value<0.0001). No episodes of aggression occurred in any of the groups.

c Design and results from the sociability test, where implanted adult virgin female mice can choose between an empty chamber and one with a juvenile virgin female conspecific present. (n=13-24 trials per group, duplicates or triplicates from 7-8 mice per group, ordinary one-way ANOVA with Tukey's test for correcting for multiple comparisons, ANOVA P value<0.0001).

d Design and results from the pup retrieval test, with implanted adult virgin female mice (n=21-24 trials per group, triplicates from 7-8 mice per group, ordinary one-way ANOVA).

e Results from the open field test, with implanted adult virgin female mice (n=21-24 trials per group, triplicates from 7-8 mice per group, ordinary one-way ANOVA with Tukey's test for correcting for multiple comparisons, ANOVA P value<0.0001).

f Results from the elevated plus maze test, with implanted adult virgin female mice (n=21-24 trials per group, triplicates from 7-8 mice per group, ordinary one-way ANOVA with Tukey's test for correcting for multiple comparisons, ANOVA P value<0.0001).

All box plots show the median (center line), 25th and 75th percentiles (box bounds), and minima/maxima (whiskers). Source data are provided as Source Data file.
