# Supplementary material for: Maternal aggression driven by the transient mobilisation of a dormant hormone-sensitive circuit
Source: Nat Commun. 2025 Sep 29;16:8553. doi: 10.1038/s41467-025-64043-4 (PMC12480473; doi:10.1038/s41467-025-64043-4)
Supplement: Supplementary file 2 — Description of Additional Supplementary Files [file 41467_2025_64043_MOESM2_ESM.pdf]

Description of Additional Supplementary Files

Video S1: Photoactivation of PMvDAT neurons triggers maternal attack.

Lactating DAT-Cre dam expressing ChR2 in PMv DAT neurons during a resident-intruder test with an adult conspecific. Minimal baseline aggression shifts to prolonged attack bouts within seconds of blue-light photostimulation.

Video S2: Lactating dam performs pup retrieval in the absence of photostimulation of PMv DAT neurons.

Behavior of a lactating DAT-Cre dam during the pup-retrieval task, expressing ChR2 in PMv DAT neurons. In the absence of photostimulation, the dam retrieves the entire litter in less than 60 seconds.

Video S3: PMv DAT neuron photoactivation impairs pup retrieval without leading to pup-directed aggression.

Behavior of the same lactating DAT-Cre dam (as in Video S2) during the pup-retrieval task, expressing ChR2 in PMv DAT neurons. In the presence of photostimulation, pup retrieval behavior was not observed or was diminished.
