# Supplementary material for: Maternal aggression driven by the transient mobilisation of a dormant hormone-sensitive circuit
Source: Nat Commun. 2025 Sep 29;16:8553. doi: 10.1038/s41467-025-64043-4 (PMC12480473; doi:10.1038/s41467-025-64043-4)
Supplement: Supplementary file 6 — Reporting Summary [file 41467_2025_64043_MOESM6_ESM.pdf]

nature portfolio

Corresponding author(s): Stefanos Stagkourakis, PhD
Christian Broberger, MD, PhD

Last updated by author(s): Aug 6, 2025

Reporting Summary

Nature Portfolio wishes to improve the reproducibility of the work that we publish. This form provides structure for consistency and transparency in reporting. For further information on Nature Portfolio policies, see our Editorial Policies and the Editorial Policy Checklist.

Statistics

For all statistical analyses, confirm that the following items are present in the figure legend, table legend, main text, or Methods section.

n/a	Confirmed	
☐	☒	The exact sample size (*n*) for each experimental group/condition, given as a discrete number and unit of measurement
☐	☒	A statement on whether measurements were taken from distinct samples or whether the same sample was measured repeatedly
☐	☒	The statistical test(s) used AND whether they are one- or two-sided<br>*Only common tests should be described solely by name; describe more complex techniques in the Methods section.*
☐	☒	A description of all covariates tested
☐	☒	A description of any assumptions or corrections, such as tests of normality and adjustment for multiple comparisons
☐	☒	A full description of the statistical parameters including central tendency (e.g. means) or other basic estimates (e.g. regression coefficient) AND variation (e.g. standard deviation) or associated estimates of uncertainty (e.g. confidence intervals)
☐	☒	For null hypothesis testing, the test statistic (e.g. F, t, r) with confidence intervals, effect sizes, degrees of freedom and P value noted<br>*Give P values as exact values whenever suitable.*
☒	☐	For Bayesian analysis, information on the choice of priors and Markov chain Monte Carlo settings
☒	☐	For hierarchical and complex designs, identification of the appropriate level for tests and full reporting of outcomes
☐	☒	Estimates of effect sizes (e.g. Cohen's *d*, Pearson's *r*), indicating how they were calculated

Our web collection on statistics for biologists contains articles on many of the points above.

Software and code

Policy information about availability of computer code

Data collection	In vitro electrophysiology data were collected in Clampex 10.8. Behavioural data were collected using IC capture cameras from imaging source, recording both top and side view. Confocal images were taken using the ZEN software from ZEISS.
Data analysis	Electrophysiology: Analyzed using OriginPro 9 (OriginLab) and ClampFit 10.2 (Molecular Devices)<br>Image analysis: Performed in ImageJ/Fiji<br>Behavioral recordings: Scored manually using the event-logging module in EthoVision XT12 (Noldus Information Technology)<br>Sociability test: Zone-based tracking and interaction times recorded using EthoVision XT12<br>Histology and imaging: For epifluorescence microscopy the Zeiss Imager M1 was used. For confocal microscopy the Zeiss LSM 800 was used.<br>Statistical analysis: Conducted using GraphPad Prism 9<br>No custom or unpublished software was used.

For manuscripts utilizing custom algorithms or software that are central to the research but not yet described in published literature, software must be made available to editors and reviewers. We strongly encourage code deposition in a community repository (e.g. GitHub). See the Nature Portfolio guidelines for submitting code & software for further information.

Data

Policy information about availability of data

All manuscripts must include a data availability statement. This statement should provide the following information, where applicable:

- Accession codes, unique identifiers, or web links for publicly available datasets
- A description of any restrictions on data availability
- For clinical datasets or third party data, please ensure that the statement adheres to our policy

The authors confirm that the data supporting the findings of this study are availble within the article and/or its supplementary materials.

Research involving human participants, their data, or biological material

Policy information about studies with human participants or human data. See also policy information about sex, gender (identity/presentation), and sexual orientation and race, ethnicity and racism.

Reporting on sex and gender	N/A
Reporting on race, ethnicity, or other socially relevant groupings	N/A
Population characteristics	N/A
Recruitment	N/A
Ethics oversight	N/A

Note that full information on the approval of the study protocol must also be provided in the manuscript.

Field-specific reporting

Please select the one below that is the best fit for your research. If you are not sure, read the appropriate sections before making your selection.

☒ Life sciences ☐ Behavioural & social sciences ☐ Ecological, evolutionary & environmental sciences

For a reference copy of the document with all sections, see nature.com/documents/nr-reporting-summary-flat.pdf

Life sciences study design

All studies must disclose on these points even when the disclosure is negative.

Sample size	Exact sample sizes are reported in the figure legends. For ex vivo electrophysiology, recordings were typically performed on 5–13 neurons per condition, sampled from 3–8 animals. For in vivo experiments, including optogenetics and behavior, group sizes generally ranged from 5–13 mice per condition. Sample sizes were based on prior studies (see Stagkourakis et al., 2018 - NatNeuro) using similar approaches and were sufficient to detect statistically significant effects with appropriate analyses.
Data exclusions	Animals were excluded when histological validation revealed incorrect viral targeting, misplaced optic fibers, or inadequate transduction of PMvDAT neurons. No data points were excluded based on behavioral performance or statistical outlier detection. All included data met the assumptions of the statistical tests applied.
Replication	All key experiments were replicated 2–3 times in independent cohorts. The results presented in this study were consistent across all replication rounds.
Randomization	No randomization was performed; animals were allocated based on phenotype (e.g., aggressive vs. non-aggressive) and experimental design requirements. Mice were pre-screened for maternal aggression and then assigned to experimental groups accordingly.
Blinding	Behavioral data collection and analysis were performed blind to experimental conditions. Anatomical data analysis was also blinded, though tissue collection was not. Electrophysiological recordings were not blinded, except for the whole-cell patch-clamp recordings presented in Figure 1, which were analyzed blind to experimental condition.

Reporting for specific materials, systems and methods

We require information from authors about some types of materials, experimental systems and methods used in many studies. Here, indicate whether each material, system or method listed is relevant to your study. If you are not sure if a list item applies to your research, read the appropriate section before selecting a response.

Materials & experimental systems

n/a	Involved in the study	
☐	☒	Antibodies
☒	☐	Eukaryotic cell lines
☒	☐	Palaeontology and archaeology
☐	☒	Animals and other organisms
☒	☐	Clinical data
☒	☐	Dual use research of concern
☒	☐	Plants

Methods

n/a	Involved in the study	
☒	☐	ChIP-seq
☒	☐	Flow cytometry
☒	☐	MRI-based neuroimaging

Antibodies

Antibodies used

Rabbit anti-NeuN (1:500 Cell Signaling, D4G40)
Chicken anti-GFP (1:500; Aves Labs, GFP-1020)
Rabbit anti-pSTAT5 Tyr694 (1:500; Cell Signaling Technology, C11C5)
Rabbit anti-c-Fos (1:200; Santa Cruz Biotechnology, sc-52)
Alexa Fluor 488-conjugated donkey anti-rabbit (1:500; Invitrogen)
Alexa Fluor 647-conjugated goat anti-rabbit (1:500; Invitrogen)

Validation

All antibodies used are commercially available and widely validated. Specificity was confirmed by expected labeling patterns in relevant brain regions, consistent with vendor documentation and prior literature. No unexpected or off-target staining was observed.

Rabbit anti-NeuN (1:500; Cell Signalling, D4G40) NeuN (D4G4O) XP® Rabbit mAb recognizes endogenous levels of total NeuN protein. This monoclonal antibody was produced by immunizing animals with recombinant protein specific to the amino terminus of human NeuN protein (RNA binding protein fox-1 homolog 3). PMID: 28848607, 28429775, 28423319, 27008987

Rabbit anti-c-fos antibody sc-52 LotG1108 This antibody was produced by immunizing rabbits with the N-terminus of c-Fos of human origin. The specificity of the AbI was validated in several mouse brain areas following open field ,object recognition and CPP test and in particular in the bilateral barrel field of the primary somatosensory cortex following whisker stimulation. In the last study, the specificity of the AbI was confirmed using another anti-c-Fos antibody (SC-253) that yielded similar results. PMID: 26136670, 25870909, 20463958

Chicken anti-GFP (1:500; Aves Labs, GFP-1020) Anti-GFP (Green Fluorescent Protein) was produced by immunization of chickens with purified recombinant green fluorescent protein (GFP) emulsified in Freund's adjuvant. The antibody was analyzed by western blot analysis and immunohistochemistry using transgenic mice expressing the GFP gene product. PMID: 28957379, 28885975, 28694334

Rabbit anti-pSTAT5 (Tyr694) antibody (Cell Signaling Technology, C11C5):
This monoclonal antibody was generated by immunizing rabbits with a synthetic phosphopeptide corresponding to residues surrounding Tyr694 of human STAT5a. It specifically detects STAT5a and STAT5b only when phosphorylated at Tyr694/699. The antibody has been validated by Cell Signaling Technology using western blotting, immunohistochemistry (IHC), and immunofluorescence (IF). Specificity has been demonstrated in mouse hypothalamic tissue following prolactin stimulation and is widely used in neuroendocrine contexts. Validation and comparable applications are shown in peer-reviewed studies using hypothalamic slices and PRL-driven pSTAT5 detection. PMID: 32763155, 25896118, 21953590

Antibody use and staining protocols were consistent with our previous studies (Stagkourakis et al., 2018, Nat. Neurosci.; Stagkourakis et al., 2020, Cell).

Animals and other research organisms

Policy information about studies involving animals; ARRIVE guidelines recommended for reporting animal research, and Sex and Gender in Research

Laboratory animals

This study involved laboratory mice (Mus musculus), including wild-type C57BL/6J (JAX mice strain) and BALB/c (BALB/cAnNCrl, strain code: 028, Charles River) strains, as well as transgenic C57BL/6J dopamine transporter (DAT)-Cre mice (Ekstrand et al., 2006) and Rosa26-lox-stop-lox-TdTomato (Ai14) reporter (The Jackson Laboratory stock 007905) reporter mice. Animals were aged 1–8 months at the time of testing.

Wild animals

No wild animals were used.

Reporting on sex

Both female and male mice were used. Virgin and lactating (multiparous) female mice served as subjects in behavioral, electrophysiological, and histological experiments. Male and female BALB/c mice were used as intruders in the resident-intruder aggression paradigm to probe maternal aggression. Sex and reproductive state were integral to the experimental design, and all data were analyzed with respect to these variables where appropriate.

Field-collected samples

This study did not involve any field-collected samples.

Ethics oversight	All procedures were approved by the Stockholms Norra Djurförsöksetiska Nämnd, and conducted in accordance with Swedish national regulations and the European Communities Council Directive 86/609/EEC.

Note that full information on the approval of the study protocol must also be provided in the manuscript.

Plants

Seed stocks	This study did not involve any seed stocks or plant material.
Novel plant genotypes	This study did not involve any plant genotypes.
Authentication	Not applicable.
